# Tumor-to-tumor metastasis of diffuse large B cell lymphoma to gastric adenocarcinoma via CXCL12 (SDF-1)/CXCR4 axis: a case report

**DOI:** 10.1186/s12876-021-01844-z

**Published:** 2021-06-29

**Authors:** Yusuke Kamihara, Sayaka Murai, Shohei Kikuchi, Akinori Wada, Jun Murakami, Nam H. Dang, Tsutomu Sato

**Affiliations:** 1 Department of Hematology, Toyama University Hospital, 2630 Sugitani, Toyama, 930-0194 Japan; 2 Second Department of Internal Medicine, University of Toyama, Toyama, Japan; 3 Division of Clinical Laboratory and Blood Center, Toyama University Hospital, Toyama, Japan; 4 Division of Hematology/Oncology, University of Florida, Gainesville, FL USA

**Keywords:** Tumor-to-tumor metastasis, Gastric adenocarcinoma, CXCL12 (SDF-1)/CXCR4 axis, Case report

## Abstract

**Background:**

Tumor-to-tumor metastasis is the rare phenomenon in which one tumor exhibits metastatic deposits from another. To the best of our knowledge, there has been no prior reported case of tumor-to-tumor metastasis of a diffuse large B cell lymphoma (DLBCL) to a primary gastric adenocarcinoma.

**Case presentation:**

A 70-year-old man presented with chest discomfort. An echocardiogram showed the presence of a right ventricular tumor. A positron emission tomogram showed multiple foci of abnormal activity in right cervical lymph nodes, cardiac wall, and stomach. A right cervical lymph node biopsy specimen revealed histological features of DLBCL. An esophagogastroduodenoscopy showed a large circumferential ulceration on the gastric body. Subsequent biopsy revealed adenocarcinoma cells surrounded by infiltrating lymphoma cells. On immunohistochemical staining, lymphoma cells were positive for CXCR4 and adenocarcinoma cells were positive for CXCL12/SDF-1. The patient was treated with six cycles of R-CHOP chemotherapy regimen, resulting in a complete remission.

**Conclusions:**

This patient’s case implies that the interaction between a chemokine and its receptor may be the underlying mechanism for the observed tumor-to-tumor metastasis. Specifically, our case would suggest an involvement of the CXCL12 (SDF-1)/CXCR4 axis in the observed metastasis of DLBCL to primary gastric adenocarcinoma.

## Background

Tumor-to-tumor metastasis is the rare phenomenon in which one tumor is involved by metastatic deposits from another [1]. The recipient tumors are either malignant or benign, with renal cell carcinoma being the most common of the malignant recipients and meningioma being the most common for benign recipients [1], whereas lung and breast cancers are common donors [2]. In this report, a diffuse large B cell lymphoma (DLBCL) metastasizing to a gastric adenocarcinoma is described. No similar cases have been found in the accessible literature. Further, as a possible mechanism of tumor-to-tumor metastasis, the involvement of both C-X-C motif chemokine ligand 12/stromal cell-derived factor-1 (CXCL12/SDF-1) and C-X-C motif chemokine receptor 4 (CXCR4) is examined.

## Case presentation

**Fig. 1:**
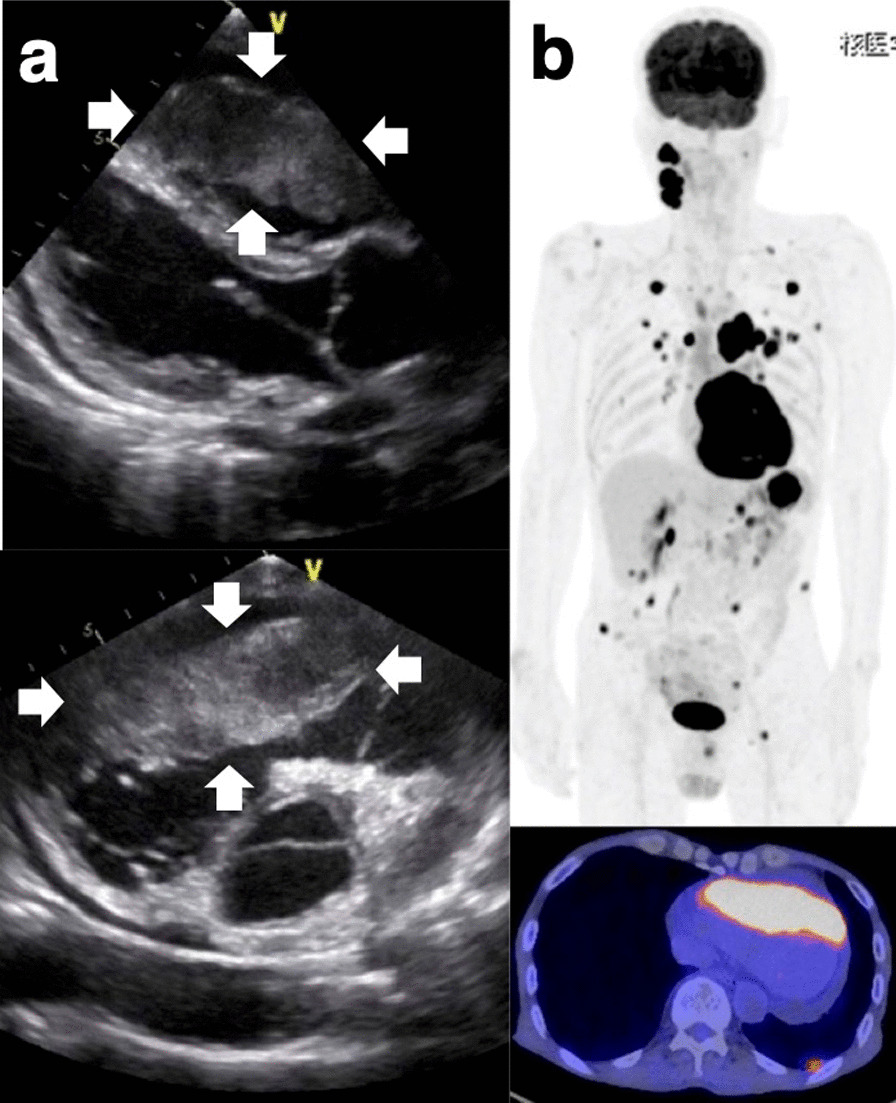
Imaging findings. **a** An echocardiogram showed the presence of a large immobile mass attached to the patient’s right ventricle extending to the outflow tract. **b** An FDG-PET showed multiple foci of abnormal FDG accumulation in right cervical and mediastinal lymph nodes, cardiac wall, and stomach

**Fig. 2:**
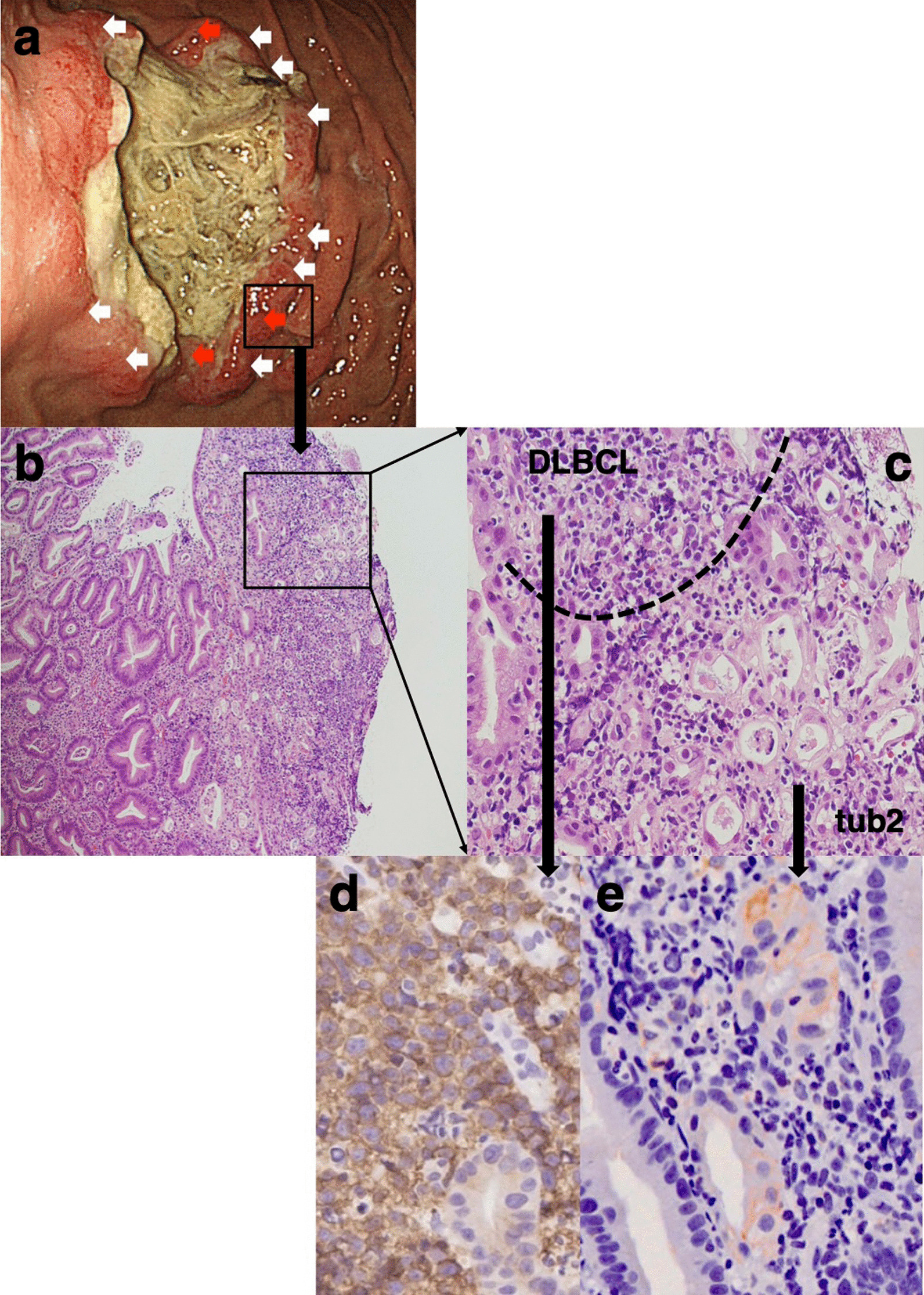
Imaging findings. **a** An EGD showed a large circumferential ulceration on the greater curvature of the gastric body. **b** Biopsy specimens from the three sites (red arrows in **a**) were found to have microtubular glands and cribriform proliferation, suggesting the existence of a moderately differentiated tubular adenocarcinoma (tub2). These adenocarcinoma cells were surrounded by DLBCL cells. **c** High-power field of **b**. **d** DLBCL cells were positive for CXCR4. **e** Adenocarcinoma cells were positive for CXCL12/SDF-1

**Fig. 3:**
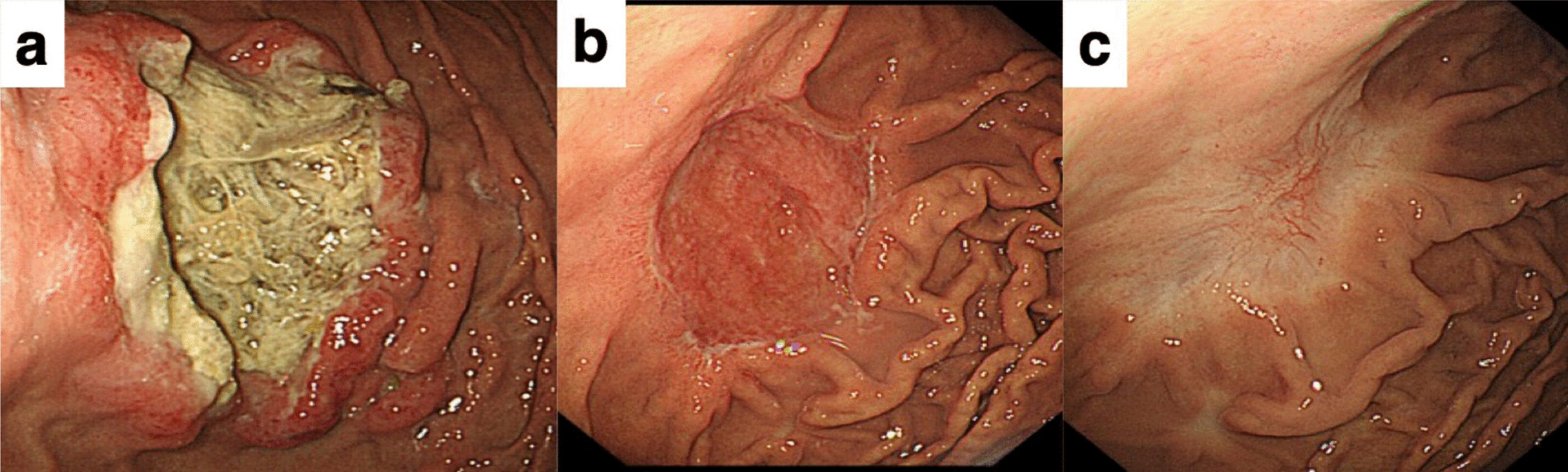
Imaging findings. Follow-up EGD after chemotherapy detected a scar lesion instead of a tumor. **a** Before the first cycle of R-CHOP chemotherapy. **b** After the third cycle. **c** After the sixth cycle

A 70-year-old man presented to his primary care physician with chest discomfort, palpitation and dizziness. He denied chest pain, shortness of breath and fatigue. When electrocardiogram revealed ST-segment elevation in all the anterior leads (V1 to V6), the patient was referred to our hospital. An echocardiogram showed the presence of a large immobile mass attached to his right ventricle extending to the outflow tract (Fig. 1a). The serum level of soluble interleukin-2 receptor was markedly increased to 6,500 U/mL (reference range, 122–496 U/mL). ^18^F-Fluorodeoxyglucose (FDG) positron emission tomogram showed multiple foci of abnormal FDG accumulation in right cervical and mediastinal lymph nodes, cardiac wall, and stomach with elevated maximum standardized uptake values of more than 27 (Fig. 1b). A right cervical lymph node biopsy specimen was obtained and revealed histological features of DLBCL, not otherwise specified, with the non-germinal center B-cell-like (non-GCB) immunophenotype, being positive for CD20, BCL6, and MUM1 and negative for CD10. An esophagogastroduodenoscopy (EGD) showed a large circumferential ulceration on the greater curvature of the gastric body (Fig. 2a). Histopathological assessment of the biopsied gastric tumor from nine different sites (white arrows in Fig. 2a) revealed diffuse infiltration of abnormal large lymphoid cells. These cells were positive for CD20, BCL-6 and MUM1 and negative for CD10. On the basis of these results, the gastric lesion was also determined to be DLBCL, non-GCB subtype. There were no pathological findings suggesting mucosa associated lymphoid tissue (MALT) lymphoma such as lymphoepithelial lesions or eosinophilic degeneration of epithelial cells. *Helicobacter pylori* (*H. pylori*) was not detected in the biopsy specimens. On the other hand, biopsy specimens from three other sites (red arrows in Fig. 2a) were found to have microtubular glands and cribriform proliferation, suggesting the existence of a moderately differentiated tubular adenocarcinoma (tub2). These adenocarcinoma cells were surrounded by DLBCL cells (Fig. 2b: low-power field, Fig. 2c: high-power field). On immunohistochemical staining using anti-human CXCR4 mouse monoclonal antibody (clone 44716) and anti-human/mouse CXCL12/SDF-1 mouse monoclonal antibody (clone 79018), DLBCL cells were positive for CXCR4 (Fig. 2d) and adenocarcinoma cells were positive for CXCL12/SDF-1 (Fig. 2e). The patient was started on R-CHOP chemotherapy regimen (rituximab, cyclophosphamide, doxorubicin, vincristine, and prednisolone), resulting in a complete remission after six cycles. Follow-up EGD after chemotherapy detected a scar lesion instead of a tumor (Fig. 3). Biopsy specimens from the scar were negative not only for lymphoma cells but also adenocarcinoma cells.

## Discussion and conclusion

The occurrence of both primary gastric lymphoma and gastric adenocarcinoma in the same patient is a rare entity. 56 cases of synchronous occurrence of both primary gastric lymphoma and gastric adenocarcinoma have been summarized by Hamaloglu et al. [3]. In their 56 cases, the majority of the lymphoma cases was MALT (69.6%) and the association of *H. pylori* with MALT was 86% in the Eastern cases and 72% in the Western cases. As for the topographic interrelation of both tumors, the majority of the cases (54.7%) had independent tumors. Collision of both tumors was reported in 14 cases (26.4%). There were 4 cases with contiguous and 5 cases with intermingling tumors.

Our case should be classified as intermingling collision tumor with *H. pylori*-negative DLBCL and adenocarcinoma in the stomach. Further, it should be noted that the gastric lymphoma in our case may not be primary but metastatic tumor from the cardiac legion since it was the largest (Fig. 1b). From this point of view, we considered the disease concept of “tumor-to-tumor metastasis, in which case the lymphoma cells had possibly metastasized into the gastric adenocarcinoma lesion, which was disrupted by rapidly growing lymphoma cells.

To the best of our knowledge, there has been no prior reported case of tumor-to-tumor metastasis of DLBCL to a primary gastric adenocarcinoma. Previously, one case of collision tumor of the stomach with both primary gastric lymphoma and adenocarcinoma was reported by Strofilas et al. [4]. They presented their case as tumor-to-tumor metastasis since the pathological examination after the gastrojejunostomy showed the focal infiltration of adenocarcinoma into lymphomatous lymph node in the perigastric area. However, this is not a true definition of tumor-to-tumor metastasis according to the criteria given by Campbell Jr et al. [5]. Campbell excluded such cases from their review of tumor-to-tumor metastasis since the metastatic region is the lymphatic systems which were already the site of lymphatic malignancies. In addition, Hu et al. [6] reported a case of simultaneous occurrence of DLBCL at the ileocecal junction and gastric intramucosal adenocarcinoma; however, they did not metastasize into each other.

As for the mechanism by which one tumor metastasizes into another, it is reasonable to implicate the involvement of specific chemokines since a particular chemokine-receptor pair can serve as tissue-specific attractant molecules for tumor cells, promoting tumor cell migration to particular sites [7–9]. For example, a potential chemokine-receptor pair was examined by immunohistochemical staining in a patient with small cell lung carcinoma (SCLC) metastasizing to renal oncocytoma (RO) [10]. The SCLC cells were positive for CXCL12/SDF-1; however, both SCLC and RC cells were negative for CXCR4.

**Fig. 4:**
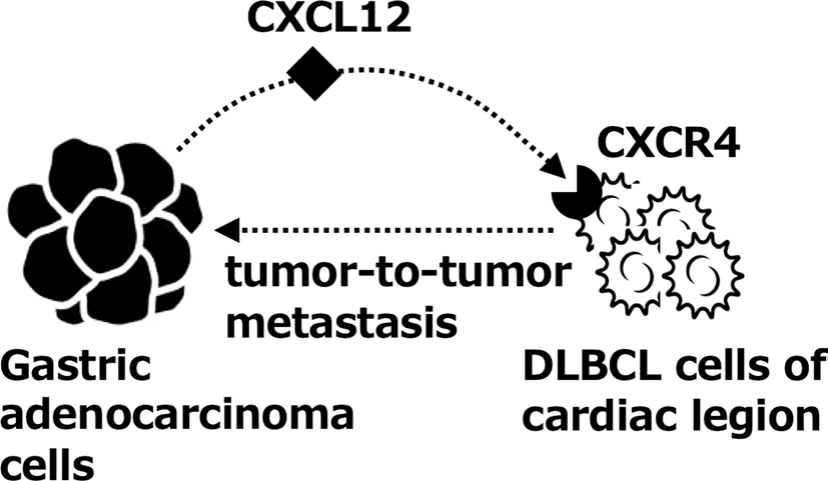
Schematic overview of possible involvement of CXCL12 (SDF-1)/CXCR4 axis in tumor-to-tumor metastasis

In this report, we successfully demonstrated the expression of both CXCL12/SDF-1 by the recipient adenocarcinoma cells and CXCR4 by the donor lymphoma cells. This is the first report which clearly suggests the possible involvement of CXCL12 (SDF-1)/CXCR4 axis in the process of tumor-to-tumor metastasis (Fig. 4). It is reported that 75.4% of DLBCL cells were positive for CXCR4 [11] and 77.3% of gastric adenocarcinoma cells were positive for CXCL12/SDF-1 [12]. These previous findings would support the rationality of our results and conclusion. Additional evidence is needed in future detailed studies to evaluate the potential pivotal role of the CXCL12 (SDF-1)/CXCR4 axis in tumor-to-tumor metastasis, along with its previously described role in tumor development, survival, angiogenesis, metastasis, and tumor microenvironment.

In summary, our case suggests the involvement of CXCL12 (SDF-1)/CXCR4 axis in tumor-to-tumor metastasis of DLBCL to primary gastric adenocarcinoma.

## Data Availability

Not applicable.

## Abbreviations

DLBCL: Diffuse large B cell lymphoma
CXCL12: C-X-C motif chemokine ligand 12
SDF-1: Stromal cell-derived factor-1
CXCR4: C-X-C motif chemokine receptor 4
FDG: ^18^F-Fluorodeoxyglucose
EGD: Esophagogastroduodenoscopy
MALT: Mucosa associated lymphoid tissue
*H. pylori*: *Helicobacter pylori*
SCLC: Small cell lung carcinoma
RO: Renal oncocytoma
non-GCB: Non-germinal center B-cell-like
